# Disclosure of plasma p‐tau217 measure improves diagnostic confidence in patients with Alzheimer's disease versus syndromes associated with frontotemporal lobar degeneration

**DOI:** 10.1002/alz.70289

**Published:** 2025-05-15

**Authors:** Valentina Cantoni, Maria Sofia Cotelli, Matteo Rota, Antonella Alberici, Ilenia Libri, Hanna Huber, Kübra Tan, Roberta Ghidoni, Sonia Bellini, Henrik Zetterberg, Kaj Blennow, Nicholas J. Ashton, Barbara Borroni

**Affiliations:** ^1^ Department of Clinical and Experimental Sciences University of Brescia Brescia Italy; ^2^ Department of Continuity of Care and Frailty ASST Spedali Civili Brescia Italy; ^3^ Department of Molecular and Translational Medicine University of Brescia Brescia Italy; ^4^ Department of Psychiatry and Neurochemistry Institute of Neuroscience and Physiology, The Sahlgrenska Academy at the University of Gothenburg Gothenburg Sweden; ^5^ German Center of Neurodegenerative Diseases (DZNE) Bonn Germany; ^6^ Molecular Markers Laboratory, IRCCS Istituto Centro San Giovanni di Dio, Fatebenefratelli Brescia Italy; ^7^ Clinical Neurochemistry Laboratory, Sahlgrenska University Hospital Gothenburg Sweden; ^8^ Wisconsin Alzheimer's Disease Research Center, University of Wisconsin School of Medicine and Public Health, University of Wisconsin–Madison Madison Wisconsin USA; ^9^ Department of Neurodegenerative Disease UCL Institute of Neurology London UK; ^10^ UK Dementia Research Institute at UCL London UK; ^11^ Hong Kong Center for Neurodegenerative Diseases Shatin Hong Kong China; ^12^ Paris Brain Institute, ICM, Pitié‐Salpêtrière Hospital, Sorbonne University Paris France; ^13^ Neurodegenerative Disorder Research Center Division of Life Sciences and Medicine and Department of Neurology Institute on Aging and Brain Disorders, University of Science and Technology of China and First Affiliated Hospital of USTC Hefei P.R. China; ^14^ Banner Sun Health Research Institute Sun City Arizona USA; ^15^ Banner Alzheimer's Institute and University of Arizona Phoenix Arizona USA

**Keywords:** Alzheimer's disease, blood‐based biomarkers, diagnostic confidence, frontotemporal lobar degeneration, plasma phosphorylated tau217

## Abstract

**INTRODUCTION:**

Further research is needed to understand the performance of plasma phosphorylated tau (p‐tau)217 in the diagnostic thinking at the individual patient level. We evaluated the incremental diagnostic value of plasma p‐tau217, expressed in terms of diagnostic confidence of Alzheimer's disease (DCAD; range 0—100).

**METHODS:**

Two hundred thirty‐two patients with dementia were included and scored in terms of DCAD in a three‐step consecutive assessment: (1) clinical work‐up, (2) clinical work‐up plus plasma p‐tau217, and (3) clinical work‐up, plasma p‐tau217, plus conventional amyloid markers. Two blinded neurologists were asked to review DCAD at each step.

**RESULTS:**

DCAD accuracy, expressed as area under the curve, significantly increased from 0.93 with clinical work‐up alone, to 0.97 with clinical work‐up plus plasma p‐tau217 (*P *= 0.01), with no further increase with the addition of conventional amyloid markers (0.99, *P *= 0.13).

**DISCUSSION:**

Plasma p‐tau217 in addition to routine assessment significantly enhances diagnostic confidence that is comparable to well‐established amyloidosis biomarkers.

**Highlights:**

- Plasma phosphorylated tau (p‐tau)217 measurements increase diagnostic confidence of Alzheimer's disease.
- Plasma p‐tau217 increases diagnostic confidence comparable to traditional markers.
- Plasma p‐tau217 dosage may be helpful in addition to routine assessment.

## 1 BACKGROUND

The development of diagnostic tools capable of accurately discriminating Alzheimer's disease (AD) from frontotemporal lobar degeneration (FTLD) has become a crucial target.^1^ Decreased levels of amyloid beta (Aβ)1‐42 in the cerebrospinal fluid (CSF) and/or increased binding of amyloid ligands visualized by positron emission tomography (PET) are the most established and validated amyloid markers, being helpful in increasing the diagnostic confidence of AD (DCAD) among clinicians.^2^ However, the invasiveness, cost, and limited accessibility of these methods have led to exploring additional tools as a promising alternative for AD diagnosis and monitoring.^3^ In this context, current efforts have been made to develop blood‐based biomarkers to replace or complement the currently available CSF and PET markers. With the advent of highly sensitive immunoassays, the development of blood‐based biomarkers has accelerated; mounting evidence now supports their clinical usefulness in increasing the diagnostic accuracy of neurodegenerative dementias and AD.^4^

Among proposed plasma biomarkers, recent evidence suggests that plasma phosphorylated tau (p‐tau)217 is able to accurately identify biological AD, comparable to CSF biomarkers, and most accurately classify amyloid and tau status compared to other plasma markers.^5^, ^6^ However, it is worth noting that, despite the excellent diagnostic accuracy at the group level, plasma p‐tau217 performance in the diagnostic thinking of clinicians at the individual patient level has not yet been tested.^7^

The above observations defined the objective of this work, aimed at evaluating whether the disclosure of plasma p‐tau217 measures may increase on DCAD. To this end, we evaluated the incremental DCAD of plasma p‐tau217 in addition to the routine clinical diagnostic work‐up in patients evaluated for cognitive impairment, compared to validated biomarkers of amyloidosis. A validation of DCAD, in terms of prediction performance of the final diagnosis, concluded the work.

## 2 METHODS

### 2.1 Participants

Patients with either probable AD^8^ or FTLD,^9^, ^10^, ^11^, ^12^, ^13^ for whom plasma biological markers were available, were consecutively recruited from the Department of Clinical and Experimental Sciences, University of Brescia, Italy. Patients with FTLD were diagnosed as behavioral variant frontotemporal dementia (bvFTD),^9^ primary progressive aphasia (PPA),^10^ corticobasal syndrome (CBS),^11^ or progressive supranuclear palsy (PSP),^12^ or frontotemporal dementia with amyotrophic lateral sclerosis (FTD‐ALS),^13^ according to current clinical criteria (see Supplementary Material in supporting information for exclusion/inclusion criteria). Demographic characteristics, family history, clinical features, and available structural brain imaging were carefully recorded at the first visit.

Each patient underwent blood sampling and plasma p‐tau217 was quantified using the commercially available ALZpath assay kit on the single molecule array (Simoa) platform (Quanterix), as previously described.^5^ The study was approved by the local ethics committee (NP2189) and was conducted in accordance with the principles of the Declaration of Helsinki and the International Conference for Harmonisation Guidelines for Good Clinical Practice.

### 2.2 Study design

Patients’ data were then anonymized, and the following information was presented to two experienced neurologists in three consecutive sections, in which they were made aware of (1) demographic characteristics, family history, clinical and neuropsychological assessment, and structural imaging data (henceforth defined as “clinical work‐up”); (2) clinical work‐up plus plasma p‐tau217 dosage; and (3) clinical work‐up and plasma p‐tau217 dosage plus conventional amyloid markers, when available (i.e., CSF total tau, p‐tau181, and Aβ1‐42 or PET amyloid).

We did not provide thresholds defining positivity/negativity of the plasma p‐tau217 but only the raw values (see Supplementary Material for further details).

On the basis of the data obtained in Section 1, the two experienced neurologists formulated their etiological diagnosis (AD vs. FTLD) and rated their confidence that cognitive impairment was due to AD on a structured scale ranging from 0% to 100% (DCAD, 0%–100%). Thus, DCAD > 50% supported an AD diagnosis, whereas DCAD < 50% supported an FTLD diagnosis (see Supplementary Material).

In cases in which the same diagnosis was reached by both raters, mean DCAD was considered. When the two raters were not concordant, a third rater was asked to assess the case. The same protocol was adopted for Sections 2 and 3, in which the two neurologists were asked to revise patients’ diagnoses and DCAD after disclosure of combined clinical work‐up along with plasma p‐tau217 (Section 2) and amyloid markers (Section 3). Moreover, the final diagnosis (i.e., AD or FTLD) was provided by the dementia expert (B.B.), who had the patients in charge and who had complete access to all the available information, such as clinical work‐up and instrumental assessments, and follow‐up visits.

**RESEARCH IN CONTEXT**

1. **Systematic review**: We performed a comprehensive review of literature using traditional (e.g., PubMed) sources to investigate the use of plasma phosphorylated tau (p‐tau)217 in increasing diagnostic confidence of Alzheimer's disease among clinicians. No available data are already available on this issue.
2. **Interpretation**: The present study demonstrates that plasma p‐tau217, in addition to routine assessment, has a significant effect on diagnostic confidence that is comparable to well‐established amyloidosis biomarkers. These biomarkers could potentially serve as non‐invasive alternatives, reducing the need for more invasive or expensive diagnostic procedures.
3. **Future directions**: Future research should include multi‐center studies with larger populations to confirm these findings and refine the algorithms of dementia diagnosis and impact on clinical practice.

### 2.3 Statistical analyses

Categorical variables were reported as percentages, whereas continuous variables were reported as means ± standard deviation (SD). Comparisons of patient characteristics across FTLD subtypes were carried out through the *t* test for continuous variables and the chi‐squared test for categorical variables.

Cross‐validated receiver operating characteristic (ROC) curves were drawn to assess the DCAD performance at each of the three consecutive assessments for discriminating between (1) AD and FTLD, (2) AD and bvFTD, and (3) AD and PPA. Comparison of AUC between each of the three consecutive assessments was performed using the DeLong test. Finally, logistic regression models were fitted to quantify the strength of the association between DCAD and the final diagnosis. Statistical significance was set at *P* value ≤0.05. Statistical analyses were performed using R version 4.4.1.

## 3 RESULTS

Two hundred thirty‐two patients were considered, and according to the final diagnosis, 78 patients were classified as AD (mean age = 69.2 ± 8.4 years; female = 47.4%) and 154 as FTLD (mean age = 64.5 ± 8.9 years; female = 42.9%). In the FTLD group, 92 patients were classified as bvFTD, 54 as PPA, 3 as CBS/PSP, and 5 as FTD‐ALS. Demographic, clinical, and biological features are reported in Table 1. Plasma p‐tau217 was significantly increased in the AD group (1.39 ± 0.78 pg/mL) compared to the FTLD group (0.39 ± 0.43 pg/mL, *P *< 0.001). According to the final diagnosis, in the AD group, DCAD progressively increased using clinical work‐up alone (67% ± 16) to clinical work‐up plus plasma p‐tau217 (78% ± 16) to clinical work‐up and plasma p‐tau217 dosage plus conventional amyloid markers (89% ± 13). Otherwise, in the FTLD group, DCAD progressively decreased considering the three above steps (28% ± 12 to 21% ± 15 and to 14% ± 15).

**TABLE 1:** Demographic, clinical, and biological features of AD and FTLD patients according to final “gold standard” diagnosis.

Variables	AD (*n* = 78)	FTLD (*n *= 154)	*P* values
Age, years	69.2 ± 8.4	64.5 ± 8.9	<0.001
Sex, female%	47.4	42.9	0.50
Age at onset, years	66.1 ± 8.5	61.2 ± 8.9	<0.001
Family history for dementia, %	13.2	39.0	<0.001
Education, years	10.2 ± 4.2	10.7 ± 4.2	0.39
MMSE	22.8 ± 4.6	21.2 ± 7.6	0.09
*Plasma marker*			
Plasma p‐tau217 (pg/mL)	1.39 ± 0.78	0.39 ± 0.43	<0.001
*DCAD*			
Clinical w‐up	67 ± 16	28 ± 12	<0.001
Clinical w‐up + p‐tau217	78 ± 16	21 ± 15	<0.001
Clinical w‐up + p‐tau217 + amyloid markers	89 ± 13	14 ± 15	<0.001

*Note*: Values are medians (interquartile ranges) for continuous variables, or percentages (raw numbers) for categorical variables.

Diagnoses were based on the final diagnosis determined after the conclusion of the diagnostic clinical and instrumental work‐up by the actual managing physicians who visited and follow‐up the patient in person.

Abbreviations: AD, Alzheimer's disease; DCAD, diagnostic confidence of Alzheimer's disease; FTLD, frontotemporal lobar degeneration; MMSE, Mini‐Mental State Examination; p‐tau, phosphorylated tau; w‐up, work‐up.

Raters’ concordance on diagnosis (AD vs. FTLD) is reported in the Supplementary Material.

**FIGURE 1:**
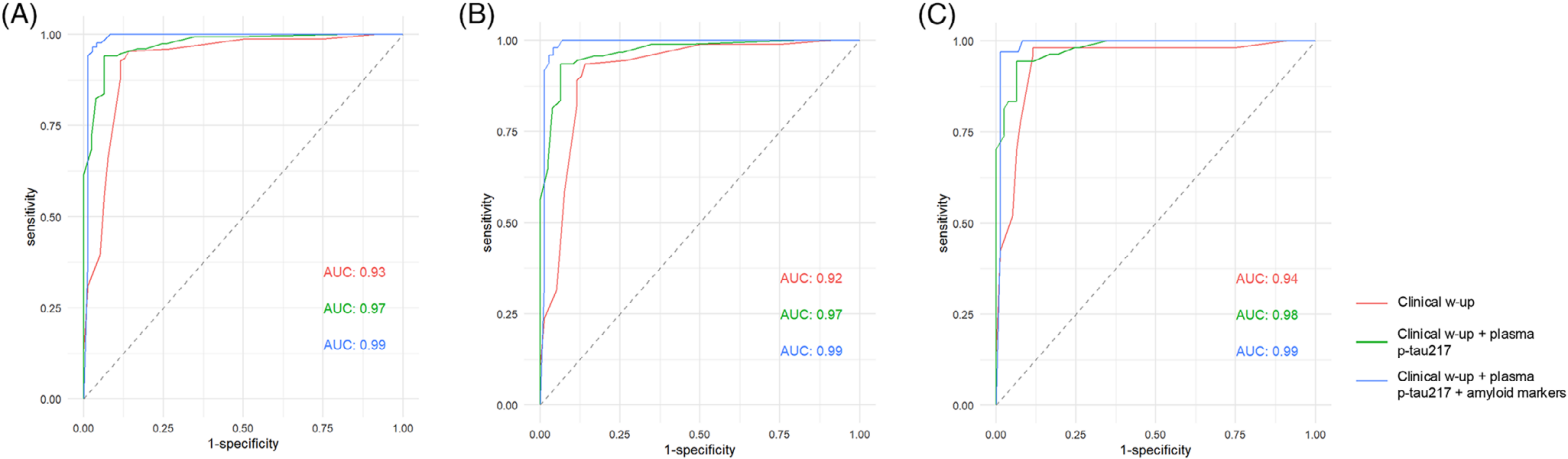
ROC curves of DCAD for clinical work‐up, clinical work‐up plus plasma p‐tau217, and clinical work‐up and p‐tau217 plus amyloid markers in differentiating AD from FTLD (A), AD from bvFTD (B), and AD from PPA (C). AD, Alzheimer's disease; AUC, area under the curve; bvFTD, behavioral variant frontotemporal dementia; clinical w‐up, clinical work‐up; DCAD, diagnostic confidence of Alzheimer's disease; FTLD, frontotemporal lobar degeneration; PPA, primary progressive aphasia; p‐tau, phosphorylated tau; ROC, receiver operating characteristic

When we applied ROC analysis to assess DCAD in the three consecutive sections carried out by neurologists blinded to final diagnosis, we observed a significant increase of DCAD from clinical work‐up (area under the curve [AUC] = 0.93, 95% confidence interval [CI] = 0.89–0.97) to clinical work‐up plus plasma p‐tau217 (AUC = 0.97, 95% CI = 0.95–0.99, *P *< 0.01), while no further significant increase was observed when conventional amyloid markers were added to clinical work‐up plus p‐tau217 (AUC = 0.99, 95% CI = 0.97–1.00, *P *= 0.13; see Figure 1A).

Compared to clinical work‐up alone, clinical work‐up plus plasma p‐tau217 improved DCAD sensitivity from 87.2% to 92.3%, while it did not affect specificity (from 93.5% to 94.1%). In other words, clinicians were able to rule out AD with high confidence (96.7%) when plasma p‐tau217 levels were low, while they did not further increase their DCAD when plasma p‐tau217 was high. We found a strong association between final diagnosis and clinical work‐up (odds ratio [OR] = 1.11, 95% CI = 1.09–1.13, *P *< 0.001), clinical work‐up plus plasma p‐tau217 (OR = 1.10, 95% CI = 1.08–1.12, *P *< 0.001), or clinical work‐up and plasma p‐tau217 plus conventional amyloid markers (OR = 1.11, 95% CI = 1.08–1.15, *P *< 0.001).

When considering bvFTD or PPA subgroups separately, we observed comparable results. DCAD significantly improved from clinical work‐up (AUC = 0.92, 95% CI = 0.87–0.96) to clinical work‐up plus plasma p‐tau217 (AUC = 0.97, 95% CI = 0.94–0.99, *P *< 0.01) in discriminating between AD and bvFTD (Figure 1B), and we observed a borderline improvement in discriminating between AD and PPA (from AUC = 0.94, 95% CI = 0.90–0.99, to AUC = 0.98, 95% CI = 0.96–1.00, *P *= 0.05, Figure 1C). No further significant increase of DCAD was obtained with the addition of conventional amyloid markers in both bvFTD and PPA (0.99, *P *= 0.18 and 0.99, *P *= 0.52).

## 4 DISCUSSION

The present pilot study aimed to resemble the future clinical practice when plasma biomarkers are available. We demonstrated that plasma p‐tau217 might support clinicians by increasing their diagnostic confidence at the individual level consistently with the final diagnosis, with values comparable to those of conventional amyloid markers. Interestingly, plasma p‐tau217 was more helpful in decreasing DCAD when its levels were low rather than increasing DCAD with high levels.

We indeed considered plasma p‐tau217, as recent literature clearly reported high diagnostic accuracy in detecting AD from the earliest disease stage, ranking it among the most promising AD‐related blood markers.^5^, ^6^ According to our pilot study, it is reasonable to assume that plasma p‐tau217 might be considered a first‐level screening/assessment of patients with cognitive complaints and therefore precede and be used as a gateway to traditional and more expensive or invasive biomarkers such as amyloid PET or CSF.

We intentionally assessed the specific performance of plasma p‐tau217 as single marker, to avoid confounds in achieving the highest diagnostic confidence considering combined biomarkers.^7^ However, defining the most robust assays (and the combination thereof) should be assessed in future studies.

Accordingly, it would be important to define guidelines to facilitate clinicians’ interpretation of plasma biomarkers to maximize their clinical utility, for example, presenting plasma biomarkers as binary (positive or negative) outcomes according to cut‐off values, or as continuous data (as we did in the present study, due to the lack of validated cut‐offs).

We acknowledge that the main limitation of this study is its retrospective and monocentric nature; it was conducted in a tertiary referral center for dementia disorders and in patients with relatively early‐onset dementia, and we cannot draw any conclusions about peripheral hospitals or primary care units. It will be of paramount importance for future studies to evaluate the performance of plasma p‐tau217 in real‐world practice.

In conclusion, our findings suggest that plasma p‐tau217 may support clinicians when added to clinical work‐up. If these results were corroborated in larger samples, plasma p‐tau217 may hold the promise to refine the algorithms of dementia diagnosis and impact clinical practice.

## AUTHOR CONTRIBUTIONS

Valentina Cantoni and Barbara Borroni contributed to the conception and design of the study; Valentina Cantoni, Maria Sofia Cotelli, Matteo Rota, Antonella Alberici, Ilenia Libri, Hanna Huber, Kübra Tan, Roberta Ghidoni, Sonia Bellini, Henrik Zetterberg, Kaj Blennow, Nicholas J. Ashton, and Barbara Borroni contributed to the acquisition and analysis of data; Valentina Cantoni, Matteo Rota, and Barbara Borroni contributed to drafting the text or preparing the figures.

## CONFLICT OF INTEREST STATEMENT

H.Z. has served on scientific advisory boards and/or as a consultant for Abbvie, Acumen, Alector, Alzinova, ALZpath, Amylyx, Annexon, Apellis, Artery Therapeutics, AZTherapies, Cognito Therapeutics, CogRx, Denali, Eisai, LabCorp, Merry Life, Nervgen, Novo Nordisk, Optoceutics, Passage Bio, Pinteon Therapeutics, Prothena, Red Abbey Labs, reMYND, Roche, Samumed, Siemens Healthineers, Triplet Therapeutics, and Wave; has given lectures sponsored by Alzecure, BioArctic, Biogen, Cellectricon, Fujirebio, Lilly, Novo Nordisk, Roche, and WebMD; and is a co‐founder of Brain Biomarker Solutions in Gothenburg AB (BBS), which is a part of the GU Ventures Incubator Program (outside submitted work). K.B. has served as a consultant and on advisory boards for Abbvie, AC Immune, ALZPath, AriBio, Beckman–Coulter, BioArctic, Biogen, Eisai, Lilly, Moleac Pte. Ltd, Neurimmune, Novartis, Ono Pharma, Prothena, Quanterix, Roche Diagnostics, Sanofi, and Siemens Healthineers; has served on data monitoring committees for Julius Clinical and Novartis; has given lectures, produced educational materials, and participated in educational programs for AC Immune, Biogen, Celdara Medical, Eisai, and Roche Diagnostics; and is a co‐founder of Brain Biomarker Solutions in Gothenburg AB (BBS), which is a part of the GU Ventures Incubator Program, outside the work presented in this paper. B.B. has served on scientific advisory boards for Alector, Alexion/Astrazeneca, AviadoBio, Lilly, Denali, Wave, and UCB. The other authors report nothing to disclose. Author disclosures are available in supporting information.

## CONSENT STATEMENT

Full written informed consent was obtained from all subjects according to the Declaration of Helsinki. The Brescia Ethics Committee approved the study protocol.

## Supporting information



Supporting Information

Supporting Information
