# Supplementary material for: Disclosure of plasma p‐tau217 measure improves diagnostic confidence in patients with Alzheimer's disease versus syndromes associated with frontotemporal lobar degeneration
Source: Alzheimers Dement. 2025 May 15;21(5):e70289. doi: 10.1002/alz.70289 (PMC12079395; doi:10.1002/alz.70289)
Supplement: Supplementary file 1 — Supporting Information [file ALZ-21-e70289-s001.docx]

**Supplementary**

***2.1 Participants.***

The reports of 260 outpatients with cognitive impairment evaluated at the Neurology Unit of the University of Brescia (Brescia, Italy), recruited from January 2016 to December 2023, were initially considered in the present study. Among them, 28 have been excluded (26 were not informative enough or inconclusive at the first visit report, and 2 was recognized by one rater despite anonymization), for a total of 232 reports included in the analyses. Final diagnosis was consistent with either Alzheimer Disease (AD),^1^ behavioural variant Frontotemporal dementia (bvFTD),^2^ Primary progressive Aphasia (PPA),^3^ Corticobasal Syndrome (CBS),^4^ or progressive Supranuclear Palsy (PSP),^5^ according to conventional clinical criteria. All included patients underwent a standardized neuropsychological evaluation and brain magnetic resonance imaging (MRI), as previously reported.^6^ Furthermore, cerebrospinal fluid (CSF) analyses (i.e., Aβ42, phosho-tau181, and total tau) or amyloid PET scan were available in 69.4% (161/232) of cases to support or rule-out AD.

Adopted inclusion criteria included: a) diagnosis of AD, bvFTD, PPA, CBS, or PSP, according to current clinical criteria;^1-5^ and b) plasma availability for p-tau217 dosage.

Exclusion criteria included: a) cerebrovascular disorders, hydrocephalus, or intra-cranial mass as documented by neuroimaging study within the past 12 months; b) a history of schizophrenia, schizoaffective disorder, delusional disorder or mood disorder with psychotic features, major depressive disorder, substance use disorder, or mental retardation according to DSM-IV criteria; c) a history of traumatic brain injury or other neurological disease; and d) significant medical problems.

***2.2 Study design.***

The preparatory activities and material described below were necessary to ensure the reproducibility of the present study.

Since the research field of blood-based biomarkers is rapidly evolving, all raters underwent a training session (lasting about 60 minutes) before the beginning of the study, consisting in the revision and discussion of the most relevant and recent papers on this topic. Furthermore, during the assessment, the raters had a form reporting the description of a sample of 35 cognitively unimpaired individuals consisting in the minimum value, median and IQR, mean and SD, and maximum value of plasma p-tau217. Importantly, we did not provide thresholds defining positivity/negativity of the plasma biomarkers but only the raw values.

The two experienced neurologists formulated their etiological diagnosis (AD vs. FTLD) and rated their confidence that cognitive impairment was due to AD (DCAD). Diagnostic confidence was rated by ticking the percentage corresponding to their appraisal of the diagnostic confidence on a visual numeric scale made of percentages organized spatially from 0 to 100%. Before the beginning of the study, the study team (including the raters) defined criteria to objectivize, as much as possible, the subjective nature of diagnostic confidence. DCAD > 50% supported an AD diagnosis, where (i) 50% corresponds to max uncertainty, (ii) 90% defines a “very high” diagnostic confidence of AD as operationalized by previous studies,^7^ and (iii) 100% corresponds to max certainty of AD diagnosis. Otherwise, DCDA < 50% supported an FTLD diagnosis, where (i) 50% corresponds to max uncertainty, (ii) 10% defines a “very high” diagnostic confidence of FTLD, and (iii) 0% corresponds to max certainty of FTLD diagnosis.

**3. Results**

***Raters’ concordance.***

The inter-rater agreement on the diagnosis (AD or FTLD) was 86.2% at T0 (200/232 concordance rate; Cronbach’s alpha = 0.83, 95% Confidence Interval [CI] 0.78 – 0.87), 89.2% at T1 (207/232 concordance rate; Cronbach’s alpha = 0.85, 95% CI 0.80 – 0.88) and 93.8% at T2 (151/161 concordance rate; Cronbach’s alpha = 0.90, 95% CI 0.87 – 0.93).

**References**

1. McKhann GM, Knopman DS, Chertkow H, et al. The diagnosis of dementia due to Alzheimer’s disease: recommendations from the National Institute on Aging-Alzheimer’s Association workgroups on diagnostic guidelines for Alzheimer’s disease. Alzheimers Dement. 2011; 7:263-269. doi:10.1016/j.jalz.2011.03.005M.
2. Rascovsky K, Hodges JR, Knopman D, et al. Sensitivity of revised diagnostic criteria for the behavioural variant of frontotemporal dementia. Brain. 2011; 134:2456-2477.
3. Gorno-Tempini ML, Hillis AE, Weintraub S, et al. Classification of primary progressive aphasia and its variants. Neurology. 2011; 76:1006- 1014.
4. Armstrong MJ, Litvan I, Lang AE, et al. Criteria for the diagnosis of corticobasal degeneration. Neurology. 2013;80:496-503.
5. Höglinger GU, Respondek G, Stamelou M, et al. Clinical diagnosis of progressive supranuclear palsy: the movement disorder society criteria. Mov Disord. 2017;32:853-864.
6. Benussi A, Karikari TK, Ashton NJ, et al. Diagnostic and prognostic value of serum NfL and p-Tau181 in frontotemporal lobar degeneration. J Neurol Neurosurg Psychiatry. 2020;91(9):960-967.
7. Altomare D, Barkhof F, Caprioglio C, et al. Clinical effect of early vs late amyloid positron emission tomography in memory clinic patients: The AMYPAD-DPMS randomized clinical trial. JAMA Neurol. 2023;80.
